# Elderly’s Attitude towards the Selected Types of e-Health

**DOI:** 10.3390/healthcare8010038

**Published:** 2020-02-13

**Authors:** Blanka Klimova, Petra Maresova, Sunwoo Lee

**Affiliations:** Faculty of Informatics and Management, University of Hradec Kralove, Rokitanského 62, 50003 Hradec Kralove, Czech Republic; blanka.klimova@uhk.cz (B.K.); petra.maresova@uhk.cz (P.M.)

**Keywords:** information and communication technology (ICT), e-Health, health service, attitude, older adults

## Abstract

This current study was sought to explore how older adults’ adaptation of information and communication technology (ICT) devices was associated with their preference for e-Health services. A total of 224 Czech older adults aged 60+ were analyzed for the study. The sample comprised 21% male and 79% female. A self-reported survey questionnaire was employed to assess the prevalence of the use of ICT devices and the Internet and general preference for e-Health services. A series of t-tests were performed between and within two groups divided into e-Health supporters and non-supporters. The results indicated that nearly half of the respondents preferred to use the Internet for searching for health-related information. We found that older adults’ use of ICT devices and educational level was significantly associated with the selection of the e-Health services. However, gender, household type, and the place for a residence did not count additional variance for the preferred e-Health services. For those who express willingness to receive the e-Health service, the preferred e-Health services should be implemented across relevant health domains. To do so, health professionals ought to provide the necessary equipment and educational programs that help older adults better access and adapt to e-Health services.

## 1. Introduction

The incorporation of information and communication technology (ICT) into the healthcare system and service, the so-called Electronic Heath (e-Health), became a substantial domain for healthcare professionals and service for the elderly [1]. The e-Health literature shows that e-Health provides easy access to and maintenance of a wide range of healthcare resources, including information and service [2,3,4]. Because older adults are more likely to cope with varied health problems [5], it is indicated that older people might be frequent users of the e-Health service, even though the dominant group of Internet users is the millennial generation [5]. De Veer et al. (2015) found that older adults were open-minded regarding the e-Health applications [6]. Research studies provide evidence that the use of ICT devices helps the elderly better understand their health and medical treatment [7], and the use of the Internet can positively contribute to the maintenance of cognitive functions among older adults [8]. In addition, the e-Health technologies can be effectively used for people with mild cognitive impairment and early dementia [9], and they may support integrated care for older people with multimorbidity [10]. Moreover, the use of ICT devices can reduce the economic and mental burdens of older adults and their family caregivers.

Although there is a growing trend towards the use of mobile phones among the older generations [11], the elderly still prefer using computers compared to mobile phones, as the most recent study by Reiners et al. [12] revealed is thanks to better technical aspects and access to the Internet. For example, more than half of the Italian and the German elderly reported they used the Internet to attain health-related information [3,4]. A Norwegian study also proved that the Internet use for health purposes in the Norwegian population had increased dramatically from 19% in 2000 to 67% in 2007 [13]. Older people gradually expand their use of e-Health services, such as receiving reminders for the scheduled visits, medication instructions, and consulting a doctor at a distance. Bujnowska-Fedak provided a list of the e-Health services that older people most used: accessing and managing personal health records, participating in forums on self-help groups focusing on health and illness, and ordering medicine or other medicinal products [5]. In their recent study, conducted among 1000 Polish citizens, Waligora and Bujnowska-Fedak [14] state that most of the people (84%) would like to receive SMS (short message service) reminders for appointments and prescribed medicines, which was then followed by e-registration (77.9%), viewing test results online (80.6%), or receiving basic medical recommendations (75.7%).

As a Swedish study [11] indicates, there are still feelings of ambivalence towards e-health among the elderly, especially in the area of mistrust in poor IT systems or impaired abilities to cope with technology. Therefore, the potential e-Health devices and their implementation should be targeted at the elderly people’s needs and preferences [12,13]. Reiners et al. [12] summarize these needs as follows:

- The e-Health devices should be personalized and tailored to the target user group;
- Easy access to the Internet should be provided;
- Family members should be involved in the use of the e-Health devices.

The rapidly increasing aging population [14], in particular, older adults living alone in Czech society, call for the incorporation of e-Health into the social and healthcare services, in order to best manage their demands on medical care. Data released from the Survey of Health and Retirement in Europe (SHARE) show that 48% of Czech adults aged 65+ reported they used the Internet in the previous week, and 52% of the respondents indicated that their computer skills are fair to excellent.

Electronic healthcare is a long-term topic and priority of the European Union. The e-Health’s mission is technological support for the provision of health services. This concept is in line with the vision of modern healthcare, based on public resources, with the aim of providing quality, affordable, cost-effective modern healthcare services. The patient is a key player in the whole system and naturally increases the pressure on his or her responsibility for his or her own health. In the Czech Republic, the already established e-Receptions or e-Walks are the first steps towards it. According to [15] e-Health, it can be a tool for more effective communication, but where providers want to communicate with each other, it is necessary to know who the future users are and respect their preferences.

Building on this, the current study was sought to provide empirical data about Czech older adults’ use of ICT devices and their attitudes towards the selected e-Health services. The findings of this study will help develop and promote the e-Health services for Czech elderly.

## 2. Methods

### 2.1. Data Collection

A non-experimental cross-sectional study design was employed. A self-reported survey questionnaire was developed to assess the participants’ attitude towards the ICT devices such as a mobile phone, a computer, and the Internet. The questionnaire was inspired by Bujnowska and Fedak [2,5], in order to compare and complement the results of the study focused on Poland [5]. Poland has undergone similar changes in terms of geographical, historical, and economic development, so comparing the results is also relevant in terms of context.

The questionnaire consisted of a total number of 14 questions categorized into four domains. The first part focused on the sociodemographic information about the respondents, including gender, age, education, and residential and household types. The second part of the questionnaire involved the information about their health condition, including medicine taken regularly and any chronic disease diagnosed. The third part of the questionnaire concerned the use of ICT, such as a mobile phone, a computer, and the Internet. The last part of the survey focused on the participants’ opinion about the use of ICT to monitor their health.

The respondents were addressed face-to-face in senior clubs, in the town of Holice and in the city of Hradec Kralove, Czech Republic. The data collection was conducted between September of 2015 and January of 2016. A total number of 270 questionnaires were distributed, and 226 questionnaires were returned (80% of the return rate). Out of 226 returned questionnaires, two questionnaires were excluded because the questionnaires were not completed. The collected data were processed electronically for a statistical analysis.

### 2.2. Data Analysis

#### 2.2.1. Subjects

The study participants were divided into two different groups: the e-Health supporter and the non-supporter. In the survey, the respondents were asked to indicate their preference for the e-Health service, using yes/no questions. A total number of six items was used to identify the e-Health supporter and the non-supporter (see Table 1). The respondent who indicated “yes” at least once across the six items was considered as the e-Health supporter (*N* = 156, 70%). Those who did not indicate any “yes” were categorized into the non-supporter group (*N* = 64). The most preferred e-Health service was telephone consultations (36%), making an appointment at the doctor online (28%), and receiving reminders for scheduled visits at the doctor via short-message service (18%).

#### 2.2.2. Data Calculation

The descriptive analysis was performed to provide the information about a sample frame. In order to compare the two groups, the e-Health supporters and non-supporters, a series of the paired sample t-test were performed. The significance level for all statistical tests was set to 0.05. Furthermore, the Person’s correlation coefficient was calculated. The following variables were selected:

“Medicine” = Do you regularly take any medicine? (scale setting 0—no, 1—yes),
“Number_tech” = How many technologies described in the questionnaire do you use?
“Acceptance” = There is a number of how many options from Table 1 the respondent would welcome. If the respondent did not say no, then this variable is 0, and if all are listed as 6,
“Wear_the_device” = setting the variable is on a scale from 1—least willing to 4—most willing,
“Smart_appliances” = willingness to use smart appliances, again scale 1 least unwilling, 4 most and last variable “CCTV”—willingness for a CCTV system where scale 1 was set least unwanted, 4 most. The IBM SPSS Statistics was used for the data analysis.

## 3. Results

### 3.1. Demographic Information about the Respondents

Table 2 displays the detailed information about the study sample and also the group differences between the e-Health supporters and the non-supporters. The sample predominantly consisted of females (79%) and 78% were aged between 65 and 74 years. Of the respondents, 68% completed the secondary education, and the majority of the sample lived in a small town up to 10,000 inhabitants (39%) or in a city with more than 100,000 inhabitants (43%). We found that there was a significant difference between the e-Health supporters and the non-supporters as far as the age and education level are concerned. Older adults, who were relatively younger (between 65 and 74 years) or/and who maintained a higher level of education, were more likely to indicate a positive response to the e-Health services. However, there was no significant difference across the gender, residential condition, and household size.

### 3.2. Information about the Drug Use and Chronic Disease among the Respondents

Table 3 provides health conditions among the respondents. Out of the respondents, 72% of the respondents reported that they regularly took drugs (*N* = 161). An average number of diseases per one respondent was 0.99 (SD = 1.0). The most common disease among the respondents was hypertension (47%), diabetes (15%), and Ischemic heart disease (11%), while 24% reported no chronic disease. There was a statistically significant difference between the e-Health supporters and the non-supporters according to their medical conditions.

### 3.3. Use of ICT Devices and the Internet

The implementation of e-Health builds on the ICT devices, such as a computer and a mobile or a smart phone. Table 4 shows the prevalence of the ICT devises among the study respondents. Of the respondents, 60% reported that they had a computer at home, and 57% reported they used a computer. Most of the respondents possessed a mobile phone, and some respondents reported they used a tablet (13%). Regarding the use of the Internet, 59% of the respondents reported they used the Internet, and 44% reported they used the Internet for health-related information. Both the e-Health supporters and the non-supporters indicated they used the mobile phone most, while the e-Health supporters were more likely to use the computer and the Internet compared to their counterpart non-supporters.

### 3.4. Telemonitoring and Smart e-Health Devices

The respondents also indicated their preference for telemonitoring, smart e-Health devices, and a camera system in their homes for the purpose of health monitoring. Table 5 shows how the respondents demonstrated their preference across the health-monitoring options. There were statistically significant differences between the e-Health supporters and the non-supporters in the measure of their attitudes towards the telemonitoring, smart e-Health devices at home, and setting the camera system in their household.

### 3.5. Preferred e-Health Services among the e-Health Supporters

In order to provide more practical insights into the promotion of the e-Health services aimed at the e-Health supporters, we included six indications of the e-Health service. The respondents were asked to mark across the indications if they would be willing to receive each e-Health service. Table 6 shows the preferred e-Health services among the e-Health supporters and how the sociodemographic information and the use of ICT were associated with each e-Health service. When the different sociodemographic variables were added to the analysis of e-Health, the age and the level of education were significantly associated with the selection of “scheduling a visit to a doctor via online”. As expected, the respondents who had a computer at home were more likely to arrange a visit to a doctor via online (61%) compared to their counterparts (6%). Those who did not have a computer at home were more likely to prefer telephone consultation (64%) compared to those who had a computer (43.4%). Similarly, the respondents who used the Internet were more likely to arrange a visit to a doctor via online (61%), while out of the respondents with no access to the Internet, only 2% selected the online reservation for the doctor visit. Thus, the respondents with no access to the Internet seemed to welcome a telephone consultation (68%) more than those with the Internet (42%).

### 3.6. Other Dependencies

Furthermore, dependency forces were searched by using a correlation coefficient. These statistics were used to find other potentially interesting dependencies. The results are shown in Table 7. The correlation coefficient showed a strong or moderate correlation between the “acceptance” variable of the readiness of the measuring device and the use of smart appliances, which can be interpreted as confirming the overall openness to technology among these respondents. The weaker dependence is shown between the level of education and the use of technology, where the link is direct. There is a weaker indirect correlation between the age and the use and adoption of these technologies. That still confirms the problem of solution, i.e., the acceptance of technology by the elderly. A certain solution is shown in providing information and education.

Taking a closer look at the dependence of education and the willingness to use technology (Table 8), it is evident that, from the level of Secondary Education I, the highest levels of grades 4 and 5 are already appearing in the willingness to accept and exploit emerging technology trends. Reluctance, or preferential column 1, does not occur at all at College and University education levels.

## 4. Discussion

The purpose of this current study was to provide the information about the prevalence of the ICT devices and the preference for the selected e-Health services among Czech older adults. The findings of our study in fact confirm and show a rising trend in the support of e-Health if compared with the finding of a similar Polish study [2]. Firstly, there was a similar number of respondents. We had a total of 224 respondents, while the Polish study contained 286 respondents. Secondly, according to the Polish study [2], 61% of the respondents had a computer at home and 19% of older individuals occasionally went online. Furthermore, 41% of the respondents were supporters of the e-Health services. Our study reported 60% of the elderly having a computer at home. However, there was a significantly higher number of those using it: 59% of the elderly use it. The number of the e-health supporters was almost the same: 44% supporters of e-Health services. Interestingly, both the Polish study [2] and our current study indicate that the female respondents were dominating, which might be connected with a longer life expectancy of females and more frequent report to the doctor for advice. In addition, the reason might be the fact that females are more positive about the devices which include health or support value, while males appreciate communication and entertainment devices [16]. The study findings showed that there were significant differences between the e-Health supporters and the non-supporters. Thirdly, younger (less than 74 years) or highly educated elderly were more likely to express positive attitudes towards the e-Health services. This was also true for the Polish study, which reported a significantly higher percentage of e-health supporters who had higher levels of education than non-supporters. Finally, both studies also showed that there was the age variation in the e-Health service [5,17]. The findings of both studies revealed that the most preferred e-Health service for all e-Health elderly people was a telephone consultation and receiving reminders for doctor’s visits. However, there was a significant difference among the younger and older elderly e-Health supporters in online reservations, which were preferred by the younger elderly.

It is interesting to report that Currie et al. [18] examined the attitudes towards the e-Health technology among older adults with chronic pain and found that those who already adapted the e-Health service were older and were more likely to live alone. Our data show that the e-Health non-supporters were more likely to report they had a disease and regularly took the drugs. The reason might be the fact that the e-Health non-supporters consisted of older elderly (aged 75 years and over). This also suggests that the attitudes or preferences for the e-Health service might change with age when there is an actual need for the formal care. In our study, however, gender, living conditions, and place of residence did not account for any additional variance for the e-Health supporters.

The results also indicated that the possession and the use of ICT devices was significantly related to a greater preference for the e-Health services. For example, almost 70% of the e-Health supporters reported they used a computer compared to their non-supporting counterparts (32%). Regarding the Internet, 70% of the e-Health supporters reported they used the Internet and also used the Internet for health information (55%). The e-Health supporters and the non-supporters were also different in their preference for the telemonitoring and smart e-Health device for households and camera system [18].

Beyond the significant difference between the two groups, our study also examined the intra-group variation among the e-Health supporters. The most preferred e-Health service, the telephone consultations, for example, was significantly associated with the respondents’ use of the computer and the Internet, while the second preferred e-Health service, making an appointment at the doctor online, was associated with the use of the computer and the Internet but also with the age and education. The remote health-monitoring service was significantly associated with all socio-demographic variables and the use of computer and the Internet. The existent e-Health literature also shows that the preferred e-Health services vary [2,19]. According to [2], for example, the online reservation at the doctor’s was a highly preferred service [2], while [19] found that making a reservation online was the least popular among older adults. All of this suggests that older adults’ attitudes towards and acceptance of the e-Health services depend on the individual’s different social and environmental conditions and nature of the service and perceived need for the service.

In sum, our findings reinforce that, despite the prevalence of ICT devices in older adults, there are still disparities in their use of technology and adoption of e-Health services. The analysis of our data showed that knowledge, skills, and possession of ICT devices are positively associated with preferred e-Health services. We further stress that age and education are the two most significant demographic factors that might affect favorable or negative attitudes towards e-Health services. Because the incorporation of the use of ICT and e-Health services for elderly care is becoming more prevalent, those older and less educated are more likely to experience digital poverty, such as a lack of information and limited access to the services. It is also important to note that only a handful of studies [20] provide information on the use of technology among Czech older adults. Therefore, our study will serve as a foundation for understanding how the use of ICT devices is associated with adoption of e-Health services among Czech older adults, respectively, in Central European countries, such as Hungary, Poland, or Slovakia.

Health professionals in medicine and healthcare should be better equipped to promote the preferred e-Health services for those who express willingness to receive the e-Health service. To do so, health professionals ought to provide the necessary equipment and educational programs that can help older adults better access and adapt the e-Health services (e.g., home-visiting service with an IT assistant). Moreover, for those older adults who demonstrate the disinclination to adapt to ICT, it is important to understand what constraints they might have. Because older adults have to learn new skills and knowledge, adaptability, competence, and self-esteem might be relevant determinants of the e-Health adaptation [17]. Promotion of the aged-friendly device and effectiveness of ICT devices for the health-related information and monitoring should be considered. This is in line with Melchiorre et al. [21], who state that advanced electronic decision support systems for physicians, self-management support of patients, and electronic systems for telemonitoring care processes are not yet widely implemented but hold the potential to improve person-centered integrated care for (older) people, especially with multiple chronic conditions.

## Figures and Tables

**Table 1:** Selected preference for the e-Health services among the respondents.

e-Health Service	
Telephone consultations	73 (36%)
Making an appointment at the doctor online	57 (28%)
Receiving reminders for scheduled visits at the doctor via short message service	37 (18%)
Receiving results of medical tests by e-mail or short message service	25 (12%)
Receiving simple medical recommendations by e-mail or short message service	6 (7%)
Remote monitoring of heath with the help of specialized device	9 (4%)

Note: Preference for the e-Health services was measured using a multiple-choice-question data analysis.

**Table 2:** Demographic profiles of the e-Health supporters and the non-supporters.

Profile	All Respondents *N* = 224	e-Health Supporters *N* = 156	Non-Supporters *N* = 68	*P*
Gender Male Female	45 (21%) 171 (79%)	32 (21%) 120 (79%)	13 (20%) 51 (80%)	0.93
Age Up to 74 75+	174 (78%) 49 (22%)	134 (86%) 22 (14%)	40 (59%) 28 (41%)	0.00
Education a Basic Secondary education I Secondary education II College education University education	32 (14%) 69 (31%) 83 (37%) 8 (4%) 31 (14%)	18 (12%) 39 (25%) 66 (42%) 7 (4%) 26 (17%)	14 (22%) 30 (45%) 17 (25%) 1 (1%) 5 (7%)	0.01
Residence Town up to 10,000 inhabitants Town up to 50,000 inhabitants City up to 100,000 inhabitants City with more than 100,000 inhabitants	125 (39%) 12 (4%) 46 (14%) 139 (43%)	86 (55%) 10 (7%) 31 (20%) 128 (82%)	39 (58%) 2 (3%) 15 (22%) 11 (16%)	0.73
Household living alone or in a specialized institute living with a partner living with a partner and children	85 (38%) 105 (48%) 30 (14%)	54 (35%) 78 (50%) 21 (15%)	31 (46%) 27 (40%) 9 (14%)	0.28

Secondary education I: secondary education without the school-leaving exam. Secondary education II: with the school-leaving exam. Note: There was a small number of missing values in demographic variables: gender (8 missing values), education (1 missing value), residence area (2 missing values), and household size (4 missing values). The missing values were treated by using a listwise deletion method throughout the analyses.

**Table 3:** Medical condition of the respondents.

Medical Condition	All Respondents *N* = 224	e-Health Supporters *N* = 156	Non-Supporters *N* = 68	*P*
Regularly taking drugs Yes No	161 (72%) 62 (28%)	105 (67%) 51 (33%)	56 (83%) 11 (16%)	0.01
Number of diseases per respondent Mean (*SD*)	0.99 (1.0)	0.88 (1.0)	1.23 (0.98)	0.02
Type of chronic disease Hypertension Ischemic heart disease Diabetes Chronic heart failure Hypothyroidism No chronic disease	96 (47%) 22 (11%) 30 (16%) 7 (4%) 12 (6%) 49 (24%)	-	-	-

**Table 4:** Use of the information and communication technology (ICT) devices and the Internet among the respondents.

Technology	All Respondents *N* = 224	e-Health Supporters *N* = 156	Non-Supporters *N* = 68	*P*
Use of ICT devices Computer Having computer at home Mobile phone Tablet	128 (57%) 135 (60%) 212 (95%) 30 (13%)	106 (68%) 109 (70%) 153 (98%) 26 (17%)	22 (32%) 26 (38%) 59 (87%) 4 (6%)	0.00
Use of Internet Using Internet for health-related information	132 (59%) 100 (44%)	109 (70%) 86 (55%)	23 (34%) 14 (21%)	0.00

**Table 5:** Preference for telemonitoring and smart e-Health devices.

Technology	All Respondents *N* = 224	e-Health Supporters *N* = 156	Non-Supporters *N* = 68	*P*
Telemonitoring	133 (64%)	109 (70%)	24 (35%)	0.01
Smart device for households	104 (51%)	87 (56%)	17 (25%)	0.01
Camera system at home	67 (31%)	50 (32%)	17 (25%)	0.04

Note: The preference indicated those who demonstrated positive attitudes (yes/somewhat yes) towards the telemonitoring and smart e-Health devices.

**Table 6:** Preference for e-Health services among the e-Health supporters (*N* = 156).

		Phone Consultations (*N* = 77)	Online Reservation (*N* = 68)	Receiving Reminders for Doctor’s Visits (*N* = 45)	Receiving Results of Medical Tests (*N* = 31)	Receiving Medical Recommendations (*N* =19)	Remote Monitoring (*N* = 11)
Gender	Male	18	17	9	9	4	1
	Female	58	51	36	22	15	10
		*P* = 0.476	*P* = 0.448	*P* = 0.944	*P* = 0.651	*P* = 0.001	*P* = 0.001
Age	Up to 74 years	65	63	36	29	18	10
	More than 75 years	13	5	9	2	1	1
		*P* = 0.357	*P* = 0.033	*P* = 0.178	*P* = 0.001	*P* = 0.001	*P* = 0.001
Education ^a^	Secondary education I	33	11	16	9	4	4
	Secondary education II	29	36	14	12	7	4
	Higher education	16	21	15	10	8	3
		*P* = 0.298	*P* = 0.001	*P* = 0.042	*P* = 0.226	*P* = 0.048	*P* = 0.001
Living status	Living alone or living in a specialized institute	31	17	18	12	5	6
	Living with a partner	39	37	19	13	9	3
	Living with (a partner) and children	8	12	7	6	5	2
		*P* = 0.314	*P* = 0.072	*P* = 0.472	*P* = 0.438	*P* = 0.217	*P* = 0.001
Residential condition	Town up to 50,000 inhabitants	54	45	26	22	12	6
	City more than 50,000 inhabitants	24	22	19	8	7	5
		*P* = 0.060	*P* = 0.242	*P* = 0.495	*P* = 0.152	*P* = 0.907	*P* = 0.001
Use of ICT device							
Computer		*P* = 0.016	*P* = 0.001	*P* = 0.550	*P* = 0.687	*P* = 0.105	*P* = 0.001
Internet		*P* = 0.003	*P* = 0.001	*P* = 0.579	*P* = 0.306	*P* = 0.146	*P* = 0.001
Internet for health info		*P* = 0.737	*P* = 0.303	*P* = 0.080	*P* = 0.547	*P* = 0.001	*P* = 0.001

**Table 7:** Interesting correlations.

Analyzed Characteristics	Age	Education	Residence	Household	Medicine	Number_Tech	Acceptance	Wear_Device	Smart_App	CCTV
Age	1	−0.224 **	0.133 *	−0.236 **	−0.344 **	−0.387 **	−0.308 **	−0.151 *	−0.129	−0.008
Education		1	−0.022	−0.017	−0.271 **	−0.330 **	−0.338 **	−0.009	−0.098	−0.072
Residence			1	−0.039	−0.122	−0.080	−0.072	−0.057	−0.073	−0.147 *
Household				1	−0.037	−0.108	−0.011	−0.025	−0.033	−0.129
Medicine					1	−0.277 **	−0.218 **	−0.117	−0.068	−0.032
number_tech						1	−0.338 **	−0.209 **	−0.194 **	−0.032
Acceptance							1	−0.352 **	−0.434 **	−0.214 **
Wear_device								1	−0.627 **	−0.217 **
Smart_app									1	−0.363 **
CCTV										1

** Correlation is significant at 0.01 level (2-tailed). * Correlation is significant at 0.05 level (2-tailed).

**Table 8:** Analysis of the dependence of education level on willingness to use ICT (all respondents).

Education		Smart_Appliances (Range)					Total
		1	2	3	4	5	
A basic education	Count	4	5	12	6	5	32
	% within Education	12.5%	15.6%	37.5%	18.8%	15.6%	100.0%
	% within Smart_appliances	22.2%	9.8%	24.0%	12.0%	9.4%	14.4%
	% of Total	1.8%	2.3%	5.4%	2.7%	2.3%	14.4%
Secondary education I	Count	9	22	7	16	15	69
	% within Education	13.0%	31.9%	10.1%	23.2%	21.7%	100.0%
	% within Smart_appliances	50.0%	43.1%	14.0%	32.0%	28.3%	31.1%
	% of Total	4.1%	9.9%	3.2%	7.2%	6.8%	31.1%
Secondary education II	Count	5	13	22	18	25	83
	% within Education	6.0%	15.7%	26.5%	21.7%	30.1%	100.0%
	% within Smart_appliances	27.8%	25.5%	44.0%	36.0%	47.2%	37.4%
	% of Total	2.3%	5.9%	9.9%	8.1%	11.3%	37.4%
College education	Count	0	2	1	1	3	7
	% within Education	0.0%	28.6%	14.3%	14.3%	42.9%	100.0%
	% within Smart_appliances	0.0%	3.9%	2.0%	2.0%	5.7%	3.2%
University education	Count	0	9	8	9	5	31
	% within Education	0.0%	29.0%	25.8%	29.0%	16.1%	100.0%
	% within Smart_appliances	0.0%	17.6%	16.0%	18.0%	9.4%	14.0%
	% of Total	0.0%	4.1%	3.6%	4.1%	2.3%	14.0%
